# Estimating the elimination feasibility in the 'end game' of control efforts for parasites subjected to regular mass drug administration: Methods and their application to schistosomiasis

**DOI:** 10.1371/journal.pntd.0006794

**Published:** 2018-11-12

**Authors:** Arathi Arakala, Christopher M. Hoover, John M. Marshall, Susanne H. Sokolow, Giulio A. De Leo, Jason R. Rohr, Justin V. Remais, Manoj Gambhir

**Affiliations:** 1 Department of Epidemiology and Preventive Medicine, Monash University, Melbourne, Australia; 2 Division of Environmental Health Sciences, School of Public Health, University of California, Berkeley, California, United States of America; 3 Division of Epidemiology and Biostatistics, School of Public Health, University of California, Berkeley, California, United States of America; 4 Department of Biology—Hopkins Marine Station, Stanford University, Pacific Grove, California, United States of America; 5 Department of Integrative Biology, University of Southern Florida, Tampa, Florida, United States of America; 6 Health Modelling and Analytics, IBM Research Australia, Melbourne, Australia; Princeton University, UNITED STATES

## Abstract

Progress towards controlling and eliminating parasitic worms, including schistosomiasis, onchocerciasis, and lymphatic filariasis, is advancing rapidly as national governments, multinational NGOs, and pharmaceutical companies launch collaborative chemotherapeutic control campaigns. Critical questions remain regarding the potential for achieving elimination of these infections, and analytical methods can help to quickly estimate progress towards—and the probability of achieving—elimination over specific timeframes. Here, we propose the *effective reproduction number*, *R*_eff_, as a proxy of elimination potential for sexually reproducing worms that are subject to poor mating success at very low abundance (positive density dependence, or Allee effects). *R*_eff_ is the number of parasites produced by a single reproductive parasite at a given stage in the transmission cycle, over the parasite’s lifetime—it is the generalized form of the more familiar basic reproduction number, *R*_*0*_, which only applies at the beginning of an epidemic—and it can be estimated in a ‘model-free’ manner by an estimator (**‘**ε’). We introduce ε, demonstrate its estimation using simulated data, and discuss how it may be used in planning and evaluation of ongoing elimination efforts for a range of parasitic diseases.

## Author summary

Critical questions remain regarding the potential for achieving elimination of helminth (worm) infections, and methods are needed to quickly estimate the probability of achieving elimination over specific timeframes. Here, we make use of methods in mathematical epidemiology to propose an estimator of elimination potential, and then apply this estimator of *R*_eff_−the number of parasites produced by a single reproductive parasite at a given stage in the transmission cycle, over the parasite’s lifetime—as a so-called ‘elimination feasibility coefficient,’ or **‘**ε.’ We compare this metric ε to commonly used metrics of population viability for the parasitic disease schistosomiasis. The value of this estimator is that it allows us to determine whether an elimination program is succeeding and how much effort might be required to bring it to completion.

## Introduction

The World Health Organization (WHO) Neglected Tropical Disease (NTD) Roadmap [1] advocates targeting several parasitic diseases, including schistosomiasis, onchocerciasis, and lymphatic filariasis (LF), for elimination or control. Control measures generally rely on mass drug administration (MDA) and environmental interventions that interrupt the parasites’ life cycles [2]. The WHO has set goals to locally eliminate (e.g. malaria from the Greater Mekong Region by 2030 [3]) or globally eradicate (e.g. yaws by 2020 [4]) certain NTDs in the coming years, and elimination may also be within reach for onchocerciasis in Guatemala [5]; Chagas disease in parts of Central and South America [5]; lymphatic filariasis in Brazil, Haiti and The Dominican Republic [5]; trachoma in Mexico [5]; and schistosomiasis in China [6].

However, assessing the control effort required to reach elimination in these settings remains a major challenge. The standard mathematical epidemiological approach relies on fitting differential equation models to epidemiological data, and examining model parameters and associated dynamical features. Such models are simple enough to be rapidly solved and yet rich enough to encode many of the mechanisms that give rise to epidemics, e.g., person-to-person contact processes, incubation times, and infectious periods [7]. However, these models require substantial knowledge of the epidemiological system so that the essential biological processes can be properly mathematically formulated. For this reason, non-parametric approaches (i.e., approaches that do not require model parameters to be fitted to data) that analyze empirical data, such as time series of infection intensity or disease prevalence, have also been proposed to answer policy-relevant questions, such as determining when a disease is close to elimination [8].

A powerful way to use mathematical epidemiology in systems in which the full model cannot be easily specified is to use quantities from well-formulated models to generate model-free analyses of commonly collected data. We refer to analyses that are ‘model-free’ as those that do not require the construction and parameterization of a mechanistic (or simulation) model.

Here, the *effective reproduction number*, *R*_eff_, is presented as one such candidate quantity for assessing the elimination feasibility of parasitic diseases. Because morbidity associated with parasitic infections is often related to infection intensity (i.e., the number or *burden* of adult parasites or worms harbored by human hosts) rather than the presence or absence of infection in a particular individual [9], the effective reproduction number is defined as the number of parasites produced by a single reproductive parasite at a given stage in the transmission cycle, over the parasite’s lifetime. The effective reproduction number is the generalized form of the more familiar basic reproduction number, *R*_*0*_, which only applies at the beginning of an epidemic. *R*_eff_ is a function of parasite population density (it should correctly be referred to as *R*_eff_(W) but we use the more compact form *R*_eff_ throughout this article) and thus changes as the parasite density changes over time [10]. When *R*_eff_ = 1, the parasite population is at an equilibrium, where it is expected to be perfectly replaced over the course of its lifetime. Furthermore, *R*_eff_ > 1 indicates a parasite population expected to increase in size while a population with *R*_eff_ < 1 would be expected to decrease.

**Fig 1:**
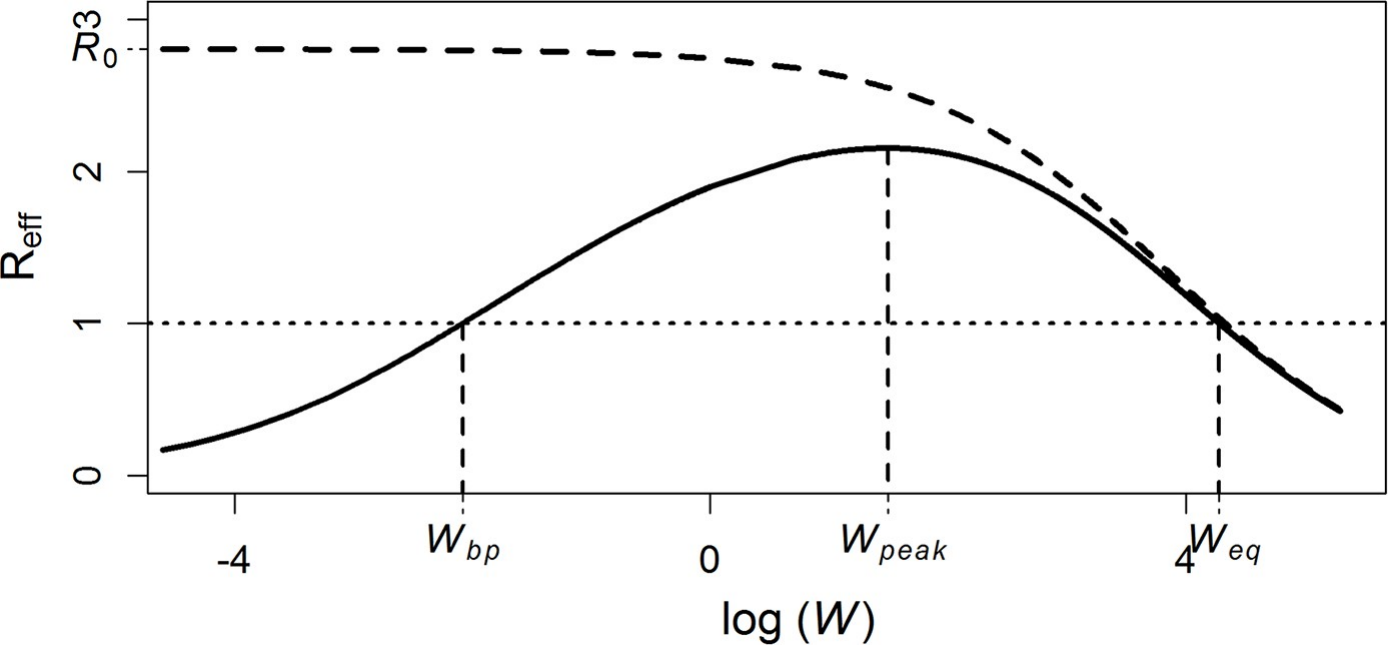
The R_eff_ profile. An *R*_eff_ profile with equilibria noted at both low (unstable; *W*_*bp*_) and high (stable; *W*_*eq*_) parasite burdens. Between these equilibria lies a point of parasite burden (*W*_*peak*_) where intense transmission is expected (transmission being proportional to the value of *R*_eff_) due to minimized influence of restrictive negative density dependence and facilitation of transmission through amplified positive density dependence. The critical influence of positive density dependence on expected values of *R*_eff_ at low worm burdens (solid line) and its deviation from expected values in the absence of positive density dependence (dashed line), where *R*_eff_ → *R*_0_, is also shown.

The *R*_eff_ profile (Fig 1) captures the relationship between *R*_eff_ and parasite population density, providing a summary of system stability across a range of parasite burdens within the human host population. Under the influence of both positive density dependence (PDD; e.g. the mating probability of dioecious—two gendered—parasites) and negative density dependence (NDD) (see [10]), the *R*_eff_ profile intersects *R*_eff_ = 1 twice and has a humped shape. Further examples of PDDs include parasite suppression of the host’s immune response [11], and of NDDs include host immunity and parasite crowding effects acting upon parasite establishment or fecundity [12]. An *endemic equilibrium* (Fig 1, *W*_*eq*_) occurs at high parasite burdens where parasite population growth is restricted by NDD (e.g., because of crowding, competition, or host immunity). The *breakpoint* (Fig 1, *W*_*bp*_) occurs at a low parasite burden where strong PDD, also known as an Allee effect [10,13], facilitates parasite elimination due to reductions in parasite reproduction (e.g., due to mate limitation) [10]. The *R*_eff_ profile can be used to estimate key features of a parasite population such as the rate of rebound following treatment, the expected parasite burden at the endemic equilibrium, or the expected breakpoint parasite population size [10]. Even though the mating probability PDD is often ignored in traditional parasitic models, there are a number of studies that have included this PDD, and breakpoints, in their theoretical analyses [10,14–18].

Below the breakpoint (Fig 1, *W*_*bp*_), *R*_eff_ < 1 and the effects of PDD are expected to drive the parasite population to elimination. The breakpoint therefore provides a logical target for intervention campaigns that decrease parasite burden, e.g., through the use of MDA, as reducing the parasite population below its breakpoint should result in elimination even without further resources devoted to intervention. Detecting the influence of PDD on parasitic disease systems and summarizing its potential influence on the elimination feasibility of sexually reproducing parasites is therefore of great value to public health practitioners developing campaigns to eliminate parasitic diseases from human populations. We hypothesize that parasite populations influenced by PDD exhibit distinct dynamics that are detectable in epidemiological datasets measuring parasite burden or prevalence through the course of an intervention campaign, and that straightforward analysis of these datasets can shed light on the elimination feasibility of parasite populations subjected to regular interventions.

To demonstrate this, the *R*_eff_ profile of the dioecious macroparasitic disease schistosomiasis is estimated, and shown to approximate the rate at which infection intensity rebounds following MDA, a quantity referred to as the Bounce Back Rate, *BBR*. Furthermore, it is shown that data collected from longitudinal intervention campaigns (e.g., annual MDA campaigns conducted in endemic schistosomiasis communities) can generate time-series of the *BBR* that can be used to develop an estimator that quantifies the elimination feasibility of the parasite population. The estimator is evaluated by comparing it to the results of commonly used population stability analyses. This estimator is presented as a simple, model-free quantity which can be used to determine the elimination feasibility of a parasitic disease subjected to regular control efforts, and more stringent and systematic collection of data that could be used to derive it is suggested.

## Methods

A mathematical model of schistosomiasis transmission, adapted from the compartmental model developed by Anderson and May [19] and from other previous work [20,21] (which focused upon *S*. *haemotobium*) is developed to describe parasite burden, measured as mean worm burden per person, *W*, in the human host population, and three disease states of the intermediate host snail population: susceptible *S*, exposed (pre-patent, *E*) and infectious (patent, *I*). The model, expressed below as Eqs 1–4, incorporates NDD via the snail population carrying capacity, *C*, acquired host immunity, *ρ*, and parasite crowding, *γ*. A PDD is implemented via the mating probability of the obligatory dioecious *Schistosoma* spp. parasites, 𝜙. The mating probability represents the probability that an individual female worm will be successfully mated, and therefore able to produce viable eggs. It is quantified as a function of the mean worm burden per person, *W*, and its distribution amongst human hosts assuming a negative binomial distribution with aggregation parameter, *κ* [22].

### Mathematical model of schistosomiasis transmission

The basic schistosomiasis model is given by the following system of differential equations:

$$\frac{dS}{dt}=f_{N}\left(1-\frac{N}{C}\right)(S+E)-\mu_{N}S-\frac{1}{2}\beta WH\phi \gamma S$$ (1)

$$\frac{dE}{dt}=\frac{1}{2}\beta WH\phi \gamma S-(\mu_{N}+\sigma )E$$ (2)

$$\frac{dI}{dt}=\sigma E-(\mu_{N}+\mu_{I})I$$ (3)

$$\frac{dW}{dt}=\lambda I\rho -(\mu_{W}+\mu_{H})W$$ (4)

where the density dependent parameters, *ϕ*, *γ*, and *ρ*, are estimated as:

$$\phi =1-\left(\frac{{\left(1-\frac{W}{W+\kappa }\right)}^{\kappa +1}}{2\pi }\int_{0}^{2\pi }\frac{1-cos\theta }{{\left(1-\frac{W}{W+\kappa }cos\theta \right)}^{\left(1+k\right)}}d\theta \right)$$ (5)

$$\gamma ={\left(1+\frac{({1-e}^{-\alpha })W}{\kappa }\right)}^{-(\kappa +1)}$$ (6)

$$\rho =e^{\left(1-vW-e^{-vW}\right)}$$ (7)

and *N* = *S* + *E* + *I* is the total snail population.

Parameter and state variable descriptions and values used in the model are shown in Table 1. The transmission parameters, *β* and *λ*, representing man-to-snail and snail-to-man transmission, respectively, are estimated by fitting the full model to epidemiological data collected in a community in the Senegal river basin as described elsewhere [20,21]. The aggregation parameter of the negative binomial distribution, *κ*, is also estimated directly from the data (S1 Text). The degree of parasite aggregation may differ for different stages of the parasite life cycle, but even complex simulation models generally use only a single aggregation parameter [25]; we therefore use a single parameter for the parasite negative binomial distribution to allow simple interpretation of our modelled results.

**Table 1:** Model parameters and state variables, their symbols, and values along with their literature basis.

Parameters	Symbol	Value	Reference
Snail fertility rate	*f*_*N*_	0.1	[20]
Snail carrying capacity	*C*	10000	[20]
Snail mortality: deaths/snail/year	*μ*_*N*_	6.2	[19]
Transition rate from exposed to infected snails (per year)	*σ*	9.1	[20]
Enhanced snail death due to infection (per year)	*μ*_*I*_	30.3	[20]
Death rate of adult worms (per year)	*μ*_*W*_	0.3	[20]
Number of human hosts	*H*	300	[20]
Death rate of humans (per year)	*μ*_*H*_	0.02	[20]
Neg Bin aggregation parameter	*Κ*	0.08	Est. from epi data
Parasite crowding density dependence parameter	*α*	0.001	[23]
Acquired immunity density dependence parameter	*v*	0.0028	[24]
Snail-to-human transmission	*λ*	1.8 × 10^−4^	Fit to epi data
Human-to-snail transmission	*β*	1.6 × 10^−6^	Fit to epi data
**State Variables**			
Susceptible snails	*S*		
Exposed snails	*E*		
Infected snails	*I*		
Adult parasite intensity per human	*W*		

#### The effective reproduction number and bounce back rate

Setting state variable time derivatives to zero in Eqs 1–3, and substituting into Eq 4, we obtain the rate of change of worm burden, expressed in terms of *R*_eff_ as:

$$\frac{dW}{dt}=W(R_{eff}-1)(\mu_{W}+\mu_{H})$$ (8)

where *R*_eff_ is given by (details in SI):

$$R_{eff}=\frac{\lambda \rho T_{1}T_{2}C(f_{N}(1+T_{2})-\mu_{N}-\beta WH\phi \gamma )}{f_{N}(1+T_{2}+T_{2}T_{1})(1+T_{2})(\mu_{W}+\mu_{H})W}$$ (9)

where $T_{1}=\frac{\sigma }{\mu_{N}+\mu_{I}}$, and $T_{2}=\frac{\frac{1}{2}\beta WH\phi \gamma }{\mu_{N}+\sigma }$. In Eq (8), *R*_*eff*_ performs the same role as the basic reproduction number *R*_0_ for the parasite dynamics close to the disease-free equilibrium (DFE) (and reduces to *R*_0_ at the DFE). We define the Bounce Back Rate, *BBR(W)* (henceforth, *BBR*), as the rate of change of parasite burden per adult parasite, expressed as:

$$BBR=\frac{1}{W}\frac{dW}{dt}$$ (10)

and therefore:

$$BBR=(R_{eff}-1)(\mu_{W}+\mu_{H})$$ (11)

The linear relationship between *R*_eff_ and *BBR*, implied by Eq 11, indicates that estimation of *BBR* can inform the estimation of *R*_eff_, thus establishing a link between the model-based *R*_eff_ and the value of *BBR* measured through field study (see below).

#### Modeling positive density dependence

To demonstrate the influence of PDD on *R*_eff_ and *BBR*, two versions of the model are simulated across a range of worm burdens, *W*, to generate and compare *R*_eff_ profiles: (1) the *PDD model* is run with *κ* = 0.08 [20] as estimated from the epidemiological data; and (2) the *PDD-free model* is run with *ϕ* = 1 implying all worms are successfully mated and therefore PDD has no influence on model dynamics.

### Estimating BBR from data

While the *BBR* can be estimated from the model (Eq 11), its value lies in its model-free estimation from empirical data. Consider a schistosomiasis intervention program in which an infected human population is treated with annual rounds of MDA. Previous to MDA in year (or other time frame) *i*, mean worm burden, *W*_*pre_i_*_, in the human population is assessed, while mean worm burden in the human population following MDA, *W*_*post_i_*_, is estimated based on the coverage of treatment and the efficacy of the administered anthelminthic drug. Therefore, in what follows, *W*_*post_i_*_ is the worm burden in the population following MDA in year *i*, and *W*_*pre_i+1_*_ is the worm burden in the human population before MDA in the following year *i+1*. Using the negative binomial aggregation parameter, *κ*, the prevalence before, $PRE{V_{pre}}_{i}$, and following, $PRE{V_{post}}_{i}$, MDA can also be estimated via simple conversion of *W* to the probability that an individual is infected. The *BBR* in year *i* is then estimated using these longitudinal infection or prevalence data as:

$$BBR_{i}=\left(\frac{1}{W_{post_{i}}}\right)\left(\frac{W_{pre_{i+1}}-W_{post_{i}}}{t_{i+1}-t_{i}}\right)$$ (12)

This is the finite-difference estimator of the continuous time *BBR* (Eq 11), which is linearly related to *R*_eff_, as previously established. Based on this relationship, it is next demonstrated that the underlying dynamics of disease transmission driven by PDD are detectable in empirical datasets by calculating the expression in Eq 12.

#### Model fitting and data generation

The model expressed as Eqs 1–4 was fitted to epidemiological data detailed elsewhere [20]. Briefly, the model is used to simulate mean worm burden in treated and untreated segments of the population which is then used to estimate the maximum likelihood transmission parameter set using the *optim* function in R [26]. A profile likelihood approach is then used to estimate the 95% confidence interval of the transmission parameters that are included in subsequent simulations. In order to focus on system dynamics that demonstrate the influence of PDD (i.e., low intensity transmission), 100 low transmission intensity parameter sets within the 95% CI were used to simulate transmission over the course of 20 years with annual MDA at 80% coverage and 94% drug efficacy—corresponding to the egg reduction ratio for *S*. *haematobium* estimated in [27]—using both the *PDD model* and the *PDD-free model*. From these simulated datasets, *W*_*pre_i_*_ and *W*_*post_i_*_ were calculated annually, allowing for the estimation of *BBR* over the course of a feasible, long-term MDA campaign, using Eq 12. Following 20 rounds of annual MDA, 40 years of intervention-free transmission are simulated to demonstrate the influence of PDD on the sustainability of worm burden reductions after an intervention campaign is halted.

### The elimination feasibility coefficient ‘ε’: Development and evaluation

From Eq 11 it can be seen that *BBR* should decrease and eventually become negative as *R*_eff_ approaches and passes the breakpoint over the course of long-term MDA. Because this behavior will only occur in the presence of strong PDD, longitudinal analysis of *BBR* can provide information on the presence and strength of PDD and the elimination feasibility of the disease. Specifically, adopting the simplest possible relationship, if *BBR* is related to time using a simple linear model:

BBR(time)=ϵ*time (13)

The parameter ε is expected to be positive, or not significantly different from zero, for the *PDD-free model*, while it should be negative for the model accounting for mating probability and PDD. The coefficient, ε fit to longitudinal *BBR* data—termed the *elimination feasibility coefficient*—is estimated using the *PDD* and *PDD-free model*s run with each of the 100 parameter sets explained above. The ability of ε to identify disease transmission dynamics that are influenced by strong PDD—which are expected to be more susceptible to elimination from MDA-based intervention—is determined by estimating ε for each model for each year 3 < *i* < 20 in the 20 years of simulated annual MDA. In addition, for each set of estimates of ε derived from the 100 parameter sets and for each year where 3 < *i* < 20, one-sided *t*-tests are used to identify significantly negative values of ε in data derived from the *PDD* and *PDD-free* models. An estimate of ε significantly less than 0 provides evidence of the influence of PDD, as it implies *BBR* is consistently decreasing with successive treatments.

The ability of ε to predict the feasibility of elimination in parasite populations influenced by PDD was evaluated by comparing ε to the probability of extinction, *P*(*e*)—or strictly-speaking *local* extinction, for parasite control programs, though we will not use the word local in what follows—a common estimate of population viability in ecology and conservation biology [28]. Derived from stochastic models of population dynamics, *P*(*e*) explicitly incorporates demographic stochasticity, known to have a strong influence on population dynamics [28]. Simulating transmission and annual MDA with a stochastic model across a range of transmission intensities 1.3x10^-4^ ≤ λ ≤ 3.0x10^-4^, corresponding to the upper and lower bounds of the 95% CI of *λ*, and parasite aggregation (0 ≤ κ ≤ 2) therefore provides an estimate of population viability in a variety of transmission scenarios. Stochastic versions of the *PDD* and *PDD-free model*s were developed using the Gillespie Stochastic Simulation Algorithm [29,30] with the *adaptivetau* package in R [31]. Additionally, random observation noise was added to simulated measurements of worm burden by resampling the estimated mean worm burden with the R function *rnbinom* to represent imperfect sampling and diagnosis associated with estimates of parasite burden in human host populations [15]. These same resampled worm burden data were then also used to estimate prevalence.

Simulated instances (*n* = 1,000) of longitudinal MDA intervention campaigns, as described in section 2.2.1, were generated for each of 2500 parameter sets drawn from the ranges of λ and κ above and holding all other parameters to values shown in Table 1. For each parameter set, the probability of extinction, *P*(*e*), was estimated as the proportion of model instances that result in elimination (i.e., induced extinction of the parasite population) out of all 1,000 instances. In each simulation, ε was estimated as in Eq 13 and the mean of all 1,000 estimates of ε for each parameter set was examined with respect to its correlation with *P*(*e*) estimates using a simple linear model with logit-transformed estimates of *P*(*e*) (thus transformed due to the natural [0,1] range of *P*(*e*)):

logit(P(e))∝ϵ (14)

Agreement between ε and *P*(*e*) implies that the model-free elimination feasibility coefficient is a reliable estimate of how likely it is the that a parasite population will be eliminated over the course of a routine MDA campaign. In summary, the higher the positive value of the elimination potential, the further from elimination the system currently lies. As the estimator becomes negative, the system is moving towards elimination and growing negative values indicate progress in this direction. In all modelled parasite prevalence and intensity trajectories that follow, we display the output values from the model unadjusted for diagnostic sensitivity or specificity.

## Results

### Influence of positive density dependence on profiles of R_eff_ and BBR

**Fig 2:**
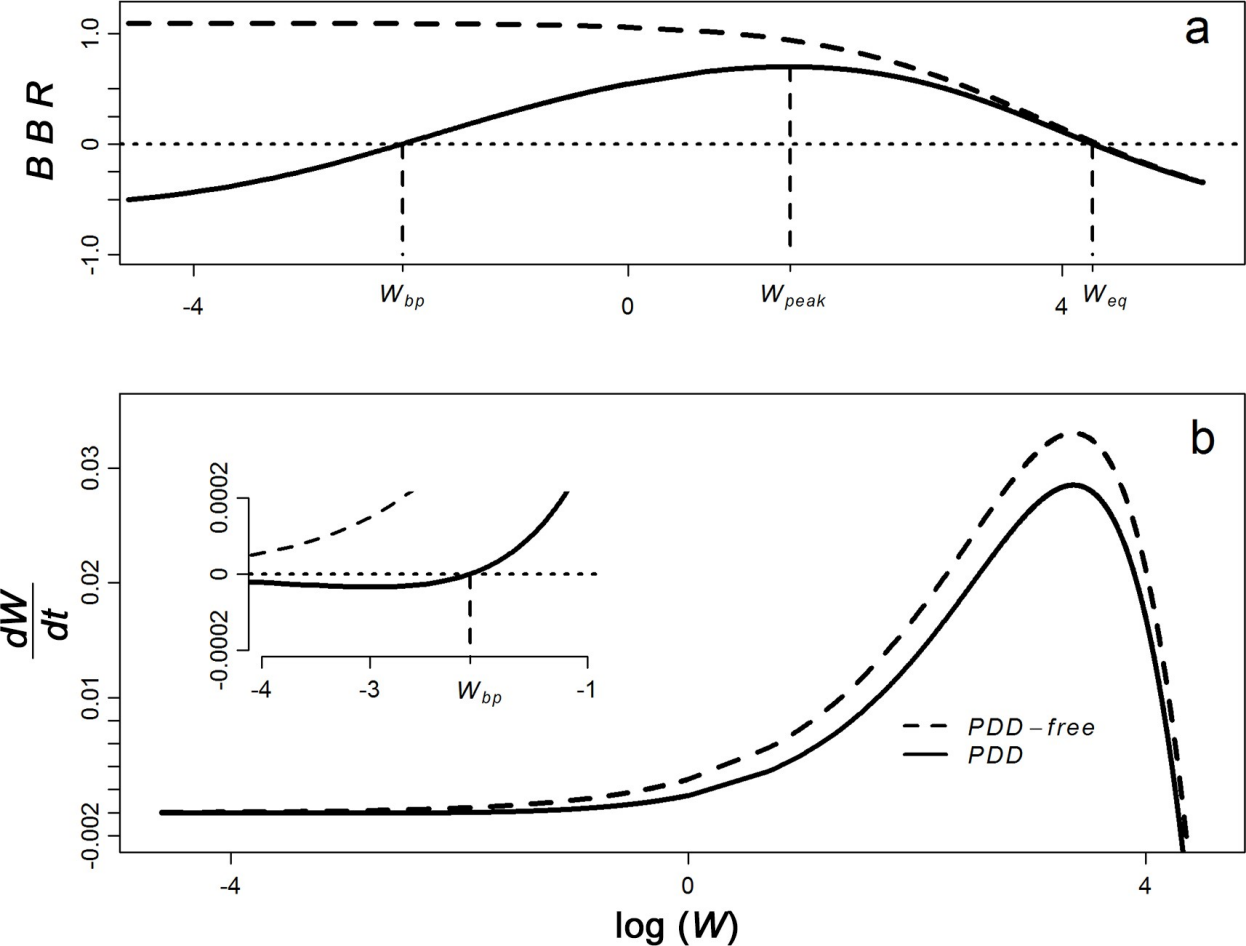
BBR profile reflecting key equilibria in relation to population parasite burden. The *BBR* profile for the *PDD* (a, solid line) and *PDD-free* (a, dashed line) *models*. Note the symmetry with the *R*_eff_ profile shown in Fig 1, including the location of key equilibria at the breakpoint (*W*_*bp*_) and endemic equilibrium (*W*_*eq*_) where *R*_eff_ and *BBR* = 0. Also shown is the rate of change of worm burden, $\frac{dW}{dt}$ (b), across log-transformed worm burden, *W*, for the *PDD* (solid line) and *PDD-free* (dashed line) models. At low values of *W* (b, inset), the models diverge as transmission in the *PDD model* (solid lines) is restricted by reduced mating probability leading to $\frac{dW}{dt}$ < 0 below the breakpoint, *W*_*bp*_.

The *R*_eff_ profile (Fig 1) indicates that, without PDD, *R*_eff_ → *R*_*0*_ as *W* → 0 (Fig 1, dashed line). The disease-free equilibrium (*W* = 0) is unstable for the *PDD-free model*, while it is always stable for the *PDD model* (Fig 2B, dashed lines). As anticipated, the *PDD model* produces a *BBR* profile with a humped shape, intersecting a line at *BBR* = 0 twice: at the breakpoint, *W*_*bp*_, and the endemic equilibrium, *W*_*eq*_ (Fig 2A, solid line). This closely resembles the *R*_eff_ profile shown in Fig 1, which can indeed be recovered from the *BBR* profile through transformation according to Eq 10. In such a system, as *W* → 0, *R*_eff_ → 0 (Fig 1A, solid line) and $\frac{dW}{dt}$ < 0 while *W* < *W*_*bp*_ and *BBR* < 0 (Fig 2A & 2B).

### Elimination feasibility coefficient from generated data

**Fig 3:**
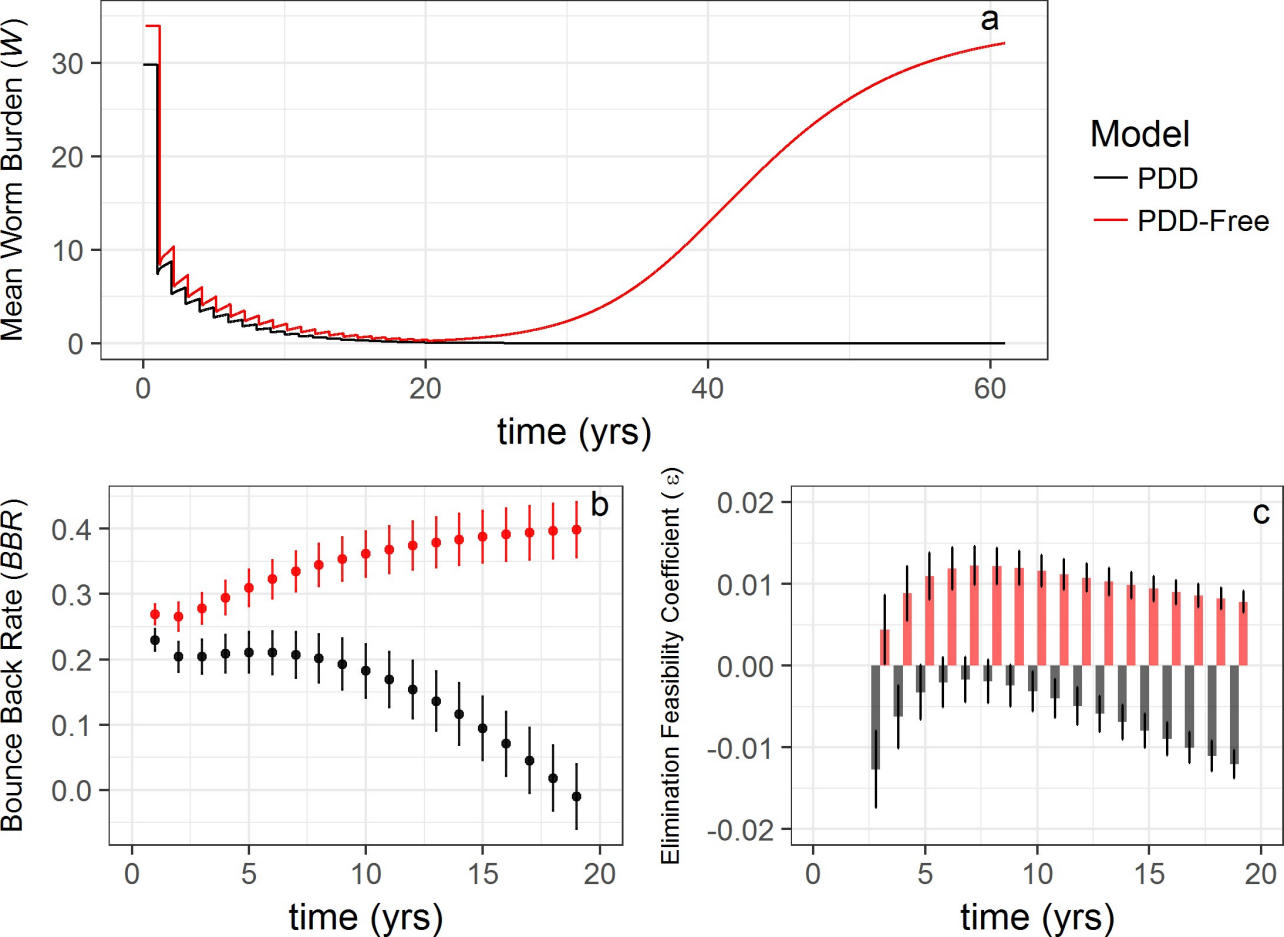
Influence of PDD on key model outputs estimated with worm burden. (a) Worm burden profiles derived from the *PDD model* (black line) and *PDD-free model* (red line) during 20 rounds of simulated annual MDA followed by 40 years with no intervention (the worm burden trajectory from the *PDD-free* model is shifted two months along the axis to improve clarity). In the presence of PDD, *W* remains at 0 even after releasing MDA as it has been suppressed below *W*_*bp*_ (a), while a lack of PDD allows *W* to rebound back to pre-MDA levels once MDA stops. (b) Mean *BBR* values of 100 model runs across 20 annual treatment rounds with error bars indicating standard deviation. (c) The elimination feasibility coefficient, ε, for the *PDD model* (black bars) and the *PDD-free model* (red bars) across each round of MDA. Error bars represent standard deviation.

A consequence of the global stability of the endemic equilibrium of the *PDD-free model* is that the parasite density will always rebound after MDA is halted [19]. In the *PDD-free model*, $\frac{dW}{dt}$ remains above 0 even with repeated MDA driving *W* → 0 (Fig 3A, red line), with the potential to rebound to pre-treatment infection levels. The low value of *W* after 20 rounds of treatment in the *PDD-free model* could mistakenly be interpreted as successful elimination, yet rebound occurs after release of treatment (Fig 3A, red line). By contrast, the *PDD model* exhibits no such rebound, since repeated MDA suppresses *W* below *W*_*bp*_ (Fig 3A, black line), restricting transmission in the absence of exogenous parasites added to the system.

With respect to bounce back rate, the *PDD model* yields a steady decline over the period of MDA and beyond caused by a decreasing mating probability as *W* → 0, and resulting in the anticipated decline in *BBR*. As *W* falls below *W*_*bp*_, a negative estimate of *BBR* is also observed (Fig 3B). The *PDD-free model* shows no such decline in *BBR* with successive MDA, as the mating probability remains high irrespective of reductions in *W*, and in fact *BBR* rises with each round of MDA as the effects of NDD diminish as *W* → 0 (Fig 3B).

The slope of the *BBR* profile provides an estimate of ε (Eq 12), the elimination feasibility coefficient and, as anticipated, the *PDD model* yields a *BBR* profile with a value of ε *<* 0, while the *PDD- free model* shows ε *>* 0. When ε *<* 0, elimination is feasible using successive MDA treatments that drive *W* below *W*_*bp*_, while ε *>* 0 implies greater resistance to achieving elimination.

**Fig 4:**
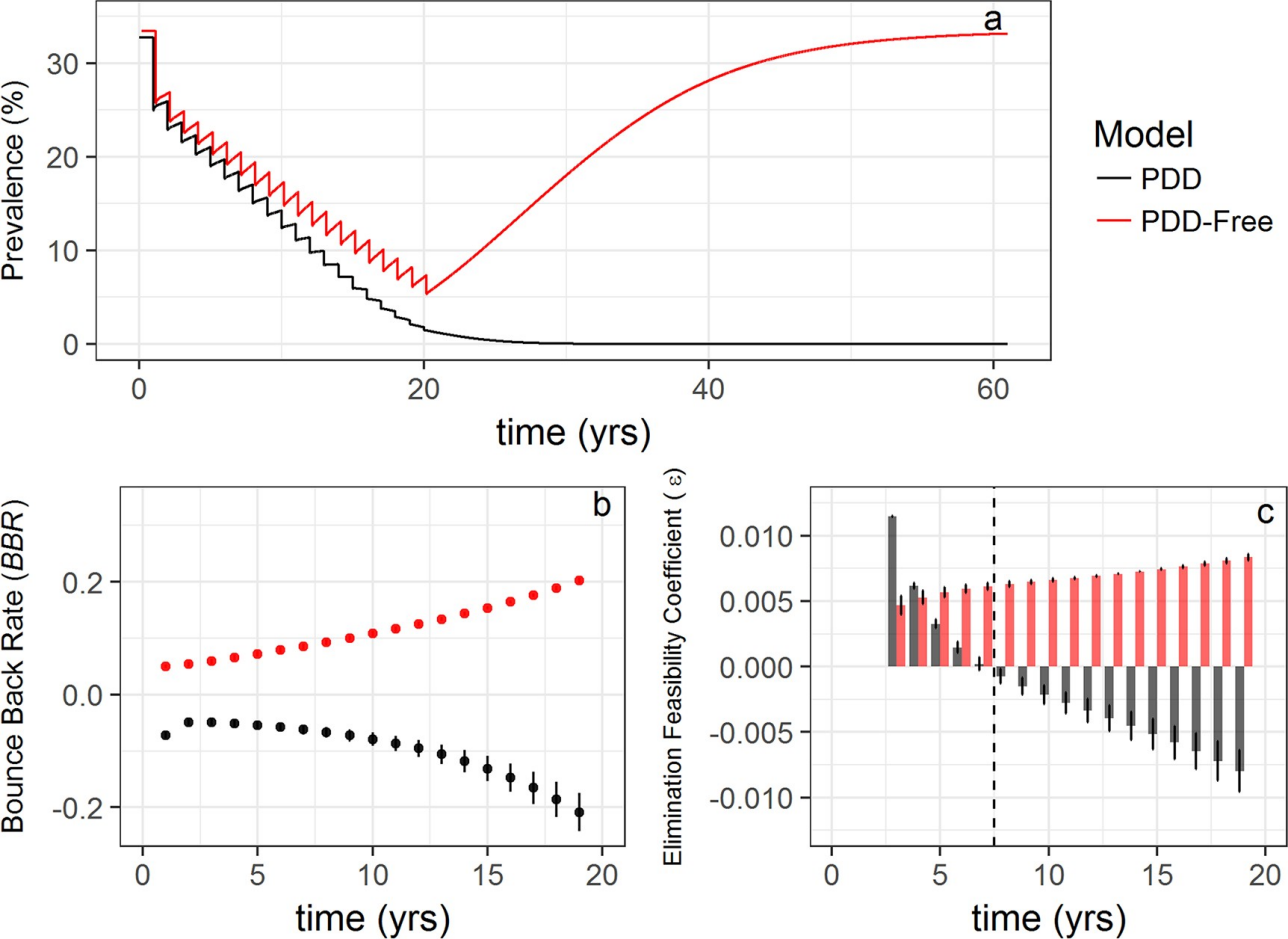
Influence of PDD on key model outputs estimated with prevalence. (a) Prevalence profiles derived from the *PDD model* (black line) and *PDD-free model* (red line) during 20 rounds of simulated annual MDA followed by 40 years with no intervention (the prevalence trajectory from the *PDD-free* model is shifted two months along the axis to improve clarity). In the presence of PDD, prevalence remains at 0% even after releasing MDA (a), while a lack of PDD allows the prevalence to rebound back to pre-MDA levels once MDA stops. (b) Mean prevalence-based *BBR* values of 100 model runs across 20 annual treatment rounds with error bars indicating standard deviation. (c) The prevalence-based elimination feasibility coefficient, ε, for the *PDD model* (black bars) and the *PDD-free model* (red bars) across each round of MDA. Error bars represent standard deviation and the dashed line between years 7 and 8 indicates the timepoint at which ε becomes significantly (p<<0) negative.

Trajectories of prevalence rather than worm burden through the course of annual MDA intervention reveals similar dynamics that yield sustainable elimination in the *PDD model* and rebound to endemic prevalence in the *PDD-free model* (Fig 4A). Prevalence based estimation of *BBR* and ε reveals two key differences from the worm burden based estimation, however. The first is that ε is initially both positive and relatively large in magnitude (Fig 4C) as a result of relatively minor changes in the overall prevalence initially induced by MDA. This leads to ε becoming negative only after 7 years of annual MDA. However, as opposed to *BBR* estimates derived from worm burden data, every estimate of the prevalence-based *BBR* is < 0 (Fig 4B) implying that, at least in the early stages, negative estimates of the prevalence-based *BBR* may serve as a better indicator of progress towards elimination than prevalence-based estimates of ε.

### Probability of extinction and elimination feasibility coefficient correlations

**Fig 5:**
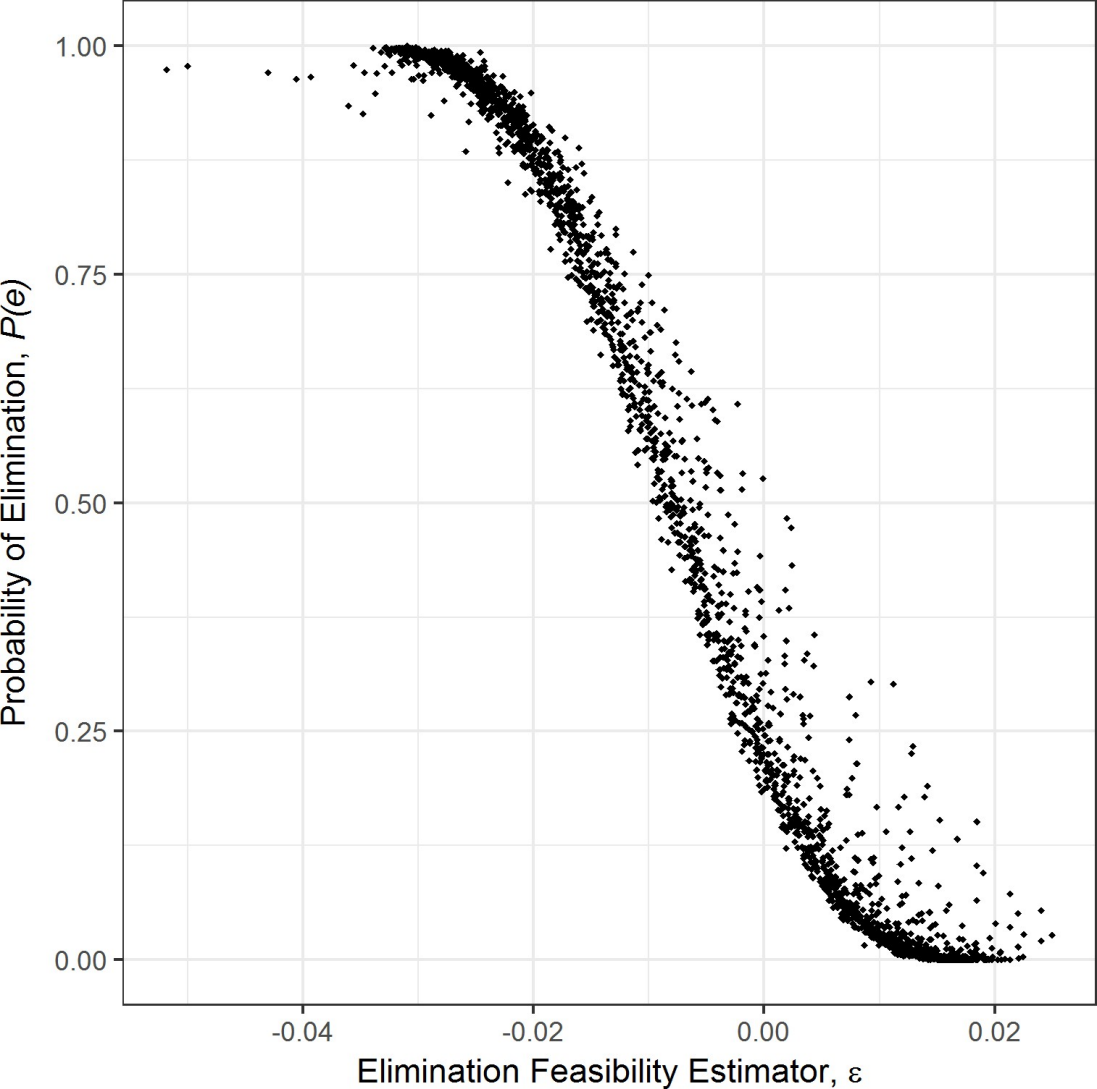
Correlation between mean ε and *P*(*e*). Correlation between mean ε and *P*(*e*) for each parameter set derived from 1,000 simulations of the stochastic model. The model-free elimination feasibility coefficient is a strong predictor of the model-predicted probability of extinction even in the presence of demographic stochasticity and observation noise (R^2^ = 0.94; probit-transformed *P*(*e*)).

The probability of extinction, *P*(*e*), was analyzed in a stochastic modeling framework across a range of realistic transmission intensities and degrees of aggregation of parasites among the human host population, which moderates the strength of PDD. A broad range of transmission scenarios were encapsulated in the tested parameter sets as *P*(*e*) ranged from 0 to 1. Fig 5 shows the mean ε for each parameter set and its corresponding value of *P*(*e*), each derived from 1,000 simulations of the stochastic model. The elimination feasibility coefficient was strongly correlated with logit-transformed *P*(*e*) (Eq 14, *R*^*2*^ = 0.94). Additionally, the proposed threshold of ε < 0 as an indicator of feasible elimination appears to be corroborated by corresponding estimates of the probability of elimination with *P*(*e*) *~ 0*.*25* when ε = 0 and *P*(*e*) → 1 as ε decreases.

### Elimination feasibility coefficient sensitivity

**Fig 6:**
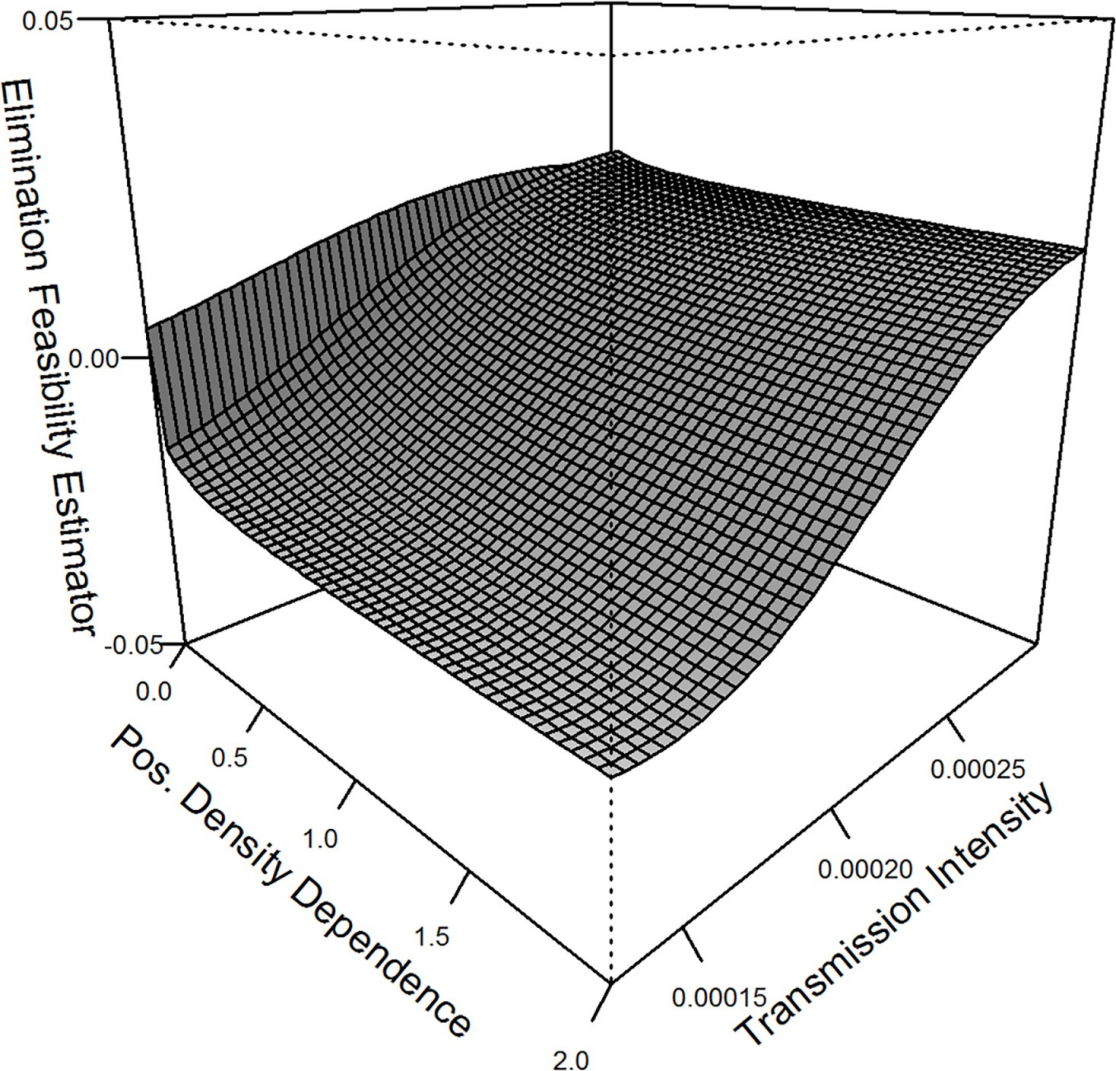
Elimination feasibility coefficient sensitivity. Sensitivity analysis showing how the elimination feasibility coefficient (ε) changes across transmission intensity, *λ*, and degree of PDD, *κ*. The surface shows a positive relationship between *λ* and ε and a negative relationship between *κ* and ε. At low transmission intensities, ε remains negative as long as κ ≠ 0 whereas high transmission intensities mostly eliminate the influence of PDD on ε, implying that annual MDA is insufficient to successfully interrupt transmission.

At low transmission intensities, the elimination feasibility coefficient remains negative in the presence of any PDD (i.e. κ > 0; Fig 6). Examining the surface resulting from estimation of ε across a range of transmission intensities and degrees of PDD illustrates that, as transmission intensity increases, ε increases and remains greater than 0 irrespective of the degree of PDD. At these high transmission intensities, single rounds of annual MDA—as were simulated in this experiment—are insufficient to make sustainable reductions in transmission.

## Discussion

Achieving local elimination of global infectious diseases, particularly parasitic worms that cause a variety of NTDs, has become a major focus of the global health community. The availability of chemotherapeutic drugs is at an historic high due to drug donations from large pharmaceutical companies, and to a commitment from multinational NGOs, such as The Bill and Melinda Gates Foundation, to combat NTDs [32]. Yet measuring and tracking progress towards elimination remains challenging [33,34].

Here, a model-free estimator of elimination potential for parasites with sexual reproduction and positive density dependence was presented, based on longitudinal measurements of worm burden or disease prevalence in human populations treated with regular MDA. The estimator, ε, is based on observations of the bounce back rate, *BBR*, of parasitic infections in the host population following MDA, which are shown to be strongly associated with the effective reproduction number, *R*_eff_. Negative values of ε derived from worm burden and/or negative values of prevalence-based *BBR* indicate consistent reductions in transmission that are expected to result in elimination if conditions such as MDA frequency and coverage hold.

Short-term estimates of *BBR* (measurable following each round of MDA) and long-term estimates of ε *(*measurable following three or more years of well-documented MDA) can provide a robust estimate of control status and progress towards elimination. Values of *BBR* derived from worm burden data that approach or reach 0 may indicate reductions in transmission that result in little to no reinfection in the measured time period. Furthermore, negative *BBR* following MDA may indicate a parasite population that has been driven below its breakpoint and is heading towards elimination due to reduced mating probabilities and below-replacement reproduction (i.e., *R*_eff_ < 1). Negative values of *BBR* derived from prevalence data may indicate successful reduction in transmission that can be coupled with longer term prevalence-based estimation of ε to indicate progress towards elimination. Meanwhile, high values of *BBR* (> 0.3) may indicate ineffective interruption of transmission and high rates of transmission (*R*_eff_ > 2).

Since most parasites that infect humans and have sexual reproduction exhibit PDD [19], ε estimates should tend to be negative as *W* → 0 in the presence of effective interventions. The elimination feasibility coefficient can therefore serve as a measure of progress and success in an intervention campaign, with negative estimates of *ε* implying a campaign is achieving sustained success in transmission interruption. On the other hand, positive values of ε could be indicative of a campaign failing to induce sustainable reductions in transmission due to unmeasured, untreated, or otherwise unaccounted for demographic groups [15], environmental conditions such as temperature and precipitation [35], and connectivity between transmission sites [36].

Sensitivity analysis of the model revealed the possibility that high, but reasonable, levels of transmission can overwhelm efforts to induce significant reductions in parasite burden using annual MDA alone. Positive estimates of ε and/or high *BBR* values could indicate such a situation, signaling especially challenging conditions for achieving elimination by MDA alone and possibly necessitating additional sanitation and/or education and/or snail control based interventions. Simultaneous estimation of *BBR* and ε can provide public health practitioners with a quantitative framework to assess the status of an NTD intervention as it transitions from morbidity control to transmission interruption to elimination.

Coupling estimates of ε and *BBR* may thus be valuable to infer disease dynamics over the course of an intervention campaign. Importantly, however, estimation of ε is reliant on consistent surveys of parasite burden (that is, measures of infection intensity rather than simple prevalence) in the human host population over multiple years. Indeed, the viability of any quantitative measure of the progress of control efforts hinges upon the data that are collected before, during, and after the campaign. Here, documentation of infection intensity in the host population prior to treatment, accurate assessment of the treatment coverage, and common assumptions regarding treatment efficacy are all necessary to derive values of *W*_*pre*_ and *W*_*pos*_ or *PREV*_*pre*_ and *PREV*_*pos*_ that are used to quantify *BBR* and ε. Such data are available from long-standing, well-documented MDA campaigns, yet rarely are these data made available in public databases [14]. It goes without saying that the collection and reporting of data on parasite burden (infection intensity) during MDA campaigns remains a critical priority, particularly as new methods become available to analyze and interpret such data with respect to estimating intervention efficacy. While infection intensity data may be preferred, we acknowledge the great difficulty in collecting parasite or egg counts from infected hosts, and demonstrate how similar methods can be adapted to more commonly and easily collected prevalence data [37].

In the present work, we examined performance of the elimination feasibility coefficient in predicting MDA success in the presence of observation noise, and under the influence of demographic stochasticity. Estimates of ε were compared to the probability of extinction, *P*(*e*), a well-established measure of population viability in conservation biology and population ecology which has recently been investigated in relation to elimination strategies in the parasitic disease Onchocerciasis [38]. A strong correlation between *P*(*e*) and ε was found, including a meaningful threshold at ε = 0, where there is approximately a 20% chance of elimination under the simulated MDA intervention scenario, and chances increase as ε decreases. These results suggest that the magnitude of the elimination feasibility coefficient may be a valuable indicator of the probability of extinction (i.e. the success of elimination efforts).

Future research should explicitly incorporate known sources of heterogeneity that may influence elimination feasibility, beyond those explored here. These should include seasonality, connected transmission sites, untreated demographic groups, and other sources of heterogeneity that can limit the impact of intervention campaigns and threaten their success. Methods to account for these sources of variability and uncertainty have been suggested, such as recent work to develop an accurate predictor of parasite extinction based upon a measure of the variance of disease prevalence over time [39]. Still other work [40] has sought to derive prevalence-based indicators of the elimination probability for STHs using an individually-based stochastic model, finding that end-point prevalence serves as the most reliable indicator of elimination probability. The work represented here adds to these analyses by proposing a potential mechanism underlying the observed effects, and providing additional approaches for estimating elimination probability. As a whole, this body of work demonstrates the utility of considering phenomena such as critical slowing down around population thresholds that is extensively investigated in other fields, but underappreciated for its applications to disease elimination. Critical slowing down is a phenomenon encountered in dynamical systems that are close to phase transitions, initially investigated in physics but carried over to ecology by Holling [41]. Since phase transitions occur in the vicinity of unstable equilibria, where the rates of change of system variables approach zero, we naturally expect this slowing down and we argue here that this is exploitable information in the context of disease elimination.

A key remaining question that deserves further theoretical and applied exploration is how to most effectively increase the breakpoint population size of sexually reproducing parasites subject to PDD, and thus increase the population’s susceptibility to elimination, especially when MDA alone appears insufficient to reach elimination due to very high transmission rates. If the breakpoint is very low, then immigration of a single infected individual from outside the treated area could result in the system suddenly returning to the trajectory towards the locally stable endemic equilibrium. Interventions that alter the degree of PDD over time by targeting high risk and/or heavily infected individuals may successfully increase the breakpoint, and interventions such as snail control, sanitation infrastructure, and education campaigns that reduce exposure may also increase the breakpoint by reducing the population’s overall capacity for transmission. The elimination feasibility coefficient, ε, represents a model-free summary estimate of the probability of elimination that can be used to evaluate ‘end game’ control programs aiming for parasite elimination.

## Supporting information

S1 Text Supporting information text file containing mathematical details of the compartmental model used in the main text and the derivation of the effective reproduction number. (PDF)
