# Supplementary material for: Estimating the elimination feasibility in the 'end game' of control efforts for parasites subjected to regular mass drug administration: Methods and their application to schistosomiasis
Source: PLoS Negl Trop Dis. 2018 Nov 12;12(11):e0006794. doi: 10.1371/journal.pntd.0006794 (PMC6258430; doi:10.1371/journal.pntd.0006794)
Supplement: S1 Text — (PDF) [file pntd.0006794.s001.pdf]

Estimating the elimination feasibility in the 'end game' of control efforts for parasites subjected to regular mass drug administration: a schistosomiasis case study - Supplementary Information

1. The compartmental model for Schistosomiasis and the Effective Reproduction Number

We adapt the basic compartmental model for the transmission of schistosomiasis by Anderson and May [19], to study the effect of interventions on the ability to eliminate the disease. The basic model of schistosomiasis is given below:

$$\frac{dS}{dt} = f_N\Big(1 - \frac{N}{C}\Big)(S+E) - \mu_N S - \frac{1}{2}\beta W H \phi\gamma S \tag{1}$$

$$\frac{dE}{dt} = \frac{1}{2}\beta W H \phi\gamma S - (\mu_N + \sigma)E \tag{2}$$

$$\frac{dI}{dt} = \sigma E - (\mu_N + \mu_I)I \tag{3}$$

$$\frac{dW}{dt} = \lambda I \rho - (\mu_W + \mu_H)W \tag{4}$$

where the density dependent parameters, Φ, γ, and ρ are estimated as:

$$\phi = 1 - \left(\frac{(1 - \frac{W}{W+\kappa})^{\kappa+1}}{2\pi}\int_0^{2\pi}\frac{1-\cos\theta}{(1 - \frac{W}{W+\kappa}\cos\theta)^{\kappa+1}}d\theta\right) \tag{5}$$

$$\gamma = \left(1 + \frac{(1-e^{-\alpha})W}{\kappa}\right)^{-(\kappa+1)} \tag{6}$$

$$\rho = e^{(1 - vW - e^{-vW})} \tag{7}$$

$\frac{dS}{dt}$, $\frac{dE}{dt}$, $\frac{dI}{dt}$ and $\frac{dW}{dt}$ represent the rate of change in the population of susceptible snails (S), exposed snails (E), infected snails (I) and adult worms (W). The description of the parameters and the values used in the model are shown in Table 1 in the main text.

1.1. The basic reproduction number and its derivation using the NGM Method

The most common epidemiological quantity estimated using mathematical modeling is the basic reproduction number R_0. *The basic reproduction number for macroparasitic diseases can be defined as the expected number of female offspring that one female parasite will produce in her lifetime* [1]. It is perhaps the most widely known measure, providing information on the epidemic growth rate and the ease or difficulty with which an infection can be controlled [10].

A common technique to estimate the value of R_0 from a compartmental model is by using the Next Generation Matrix [2, 3]. Using this method, the value of R_0 is estimated as the spectral radius of the transmission matrix $\mathbf{T}$ where $\mathbf{T}$ is constructed from the equations of the model that relate to the infectious classes. In our model, the following equations relate to the infectious class.

$$\frac{dE}{dt} = \frac{1}{2}\beta W H \phi \gamma N - (\mu_N + \sigma)E \tag{8}$$

$$\frac{dI}{dt} = \sigma E - (\mu_N + \mu_I)I \tag{9}$$

$$\frac{dW}{dt} = \lambda I \rho - (\mu_W + \mu_H)W \tag{10}$$

The coefficients of the system variables represent either the birth of new infections into the system or the transfer of infections between compartments. When the equations are represented in vector form, we can divide the right hand side into 2 matrices: F_i, the component that introduces new infections into the system and V_i, the component that represents the transfer of disease between compartments, such that $\mathbf{X} = \mathbf{F} - \mathbf{V}$.

$$\mathbf{X} = \begin{bmatrix} \frac{dE}{dt} \\ \frac{dI}{dt} \\ \frac{dW}{dt} \end{bmatrix} \tag{11}$$

$$F_i = \begin{bmatrix} \frac{1}{2}\beta WHN \\ 0 \\ \lambda I \end{bmatrix} \tag{12}$$

$$V_i = \begin{bmatrix} (\mu_N + \sigma)E \\ -\sigma E + (\mu_N + \mu_I)I \\ (\mu_W + \mu_H)W \end{bmatrix} \tag{13}$$

The transmission matrix is defined as:

$$\mathbf{T} = \mathbf{F} \times \mathbf{V}^{-1}, \tag{14}$$

where $\mathbf{F}$ and $\mathbf{V}$ are square matrices obtained by taking the partial derivative of F_i and V_i with respect to E, I and W.

$$\mathbf{F} = \begin{bmatrix} 0 & 0 & \frac{1}{2}\beta HN \\ 0 & 0 & 0 \\ 0 & \lambda & 0 \end{bmatrix} \tag{15}$$

$$\mathbf{V} = \begin{bmatrix} (\mu_N + \sigma) & 0 & 0 \\ -\sigma & (\mu_N + \mu_I)I & 0 \\ 0 & 0 & (\mu_W + \mu_H) \end{bmatrix} \tag{16}$$

This gives $\mathbf{T}$ as:

$$\mathbf{T} = \begin{bmatrix} 0 & 0 & \frac{\frac{1}{2}\beta HN}{\mu_W + \mu_H} \\ 0 & 0 & 0 \\ \frac{\sigma\lambda}{(\mu_N+\sigma)(\mu_N+\mu_I)} & \frac{\lambda}{\mu_N+\mu_I} & 0 \end{bmatrix} \tag{17}$$

R_0 is the largest Eigen value of $\mathbf{T}$ and is given by:

$$R_0 = \sqrt{\frac{\frac{1}{2}\beta H N \sigma \lambda}{(\mu_N + \sigma)(\mu_N + \mu_I)(\mu_W + \mu_H)}} \tag{18}$$

where N is the total number of susceptible snails at disease free equilibrium. In addition, we don't consider any density dependence, setting $\phi = \gamma = \rho = 1$ as these functions are not relevant at a disease free state. R_0 is a measure of disease spread within a completely susceptible population and is therefore inadequate when the interest lies in studying an established pathogen in a setting where density dependence within the pathogen population regulates transmission.

1.2. The Effective Reproduction Number

Populations of dioecious obligatory parasites are regulated by two density-dependent population regulation effects: negative density dependence or limitation on parasite fecundity and establishment, and positive density dependence or facilitation of parasite growth[12] [4]. Mathematical modeling studies have shown that positive density dependence leads to ecological *Allee effects*, a feature that can benefit elimination programs such as those for lymphatic filariasis [10] and onchocerciasis [5] .

Positive density dependence decreases the growth rate of parasites as the numbers of parasites decrease, yielding a population density threshold below which extinction could occur[6, 7]. This parasite burden level is often referred to as the *breakpoint*, below which there will not be enough parasites in the population for disease transmission to occur [7].

A macroparasitic system that shows an ecological Allee effect has two stable levels of parasite burden - a disease free one (no parasites) and an endemic one (a finite level of average parasites per host), separated by an unstable equilibrium at the breakpoint. In such an infectious disease system, the infection growth rate is zero near the disease-free equilibrium [8], but endemic disease can be established if infection levels rise above the breakpoint. For schistosomiasis, positive density dependence manifests in the worm mating probability of the dioecious worms as described in Equation 5.

Efforts to controls schistosomiasis have been focussed on MDA with the drug praziquantel. However in areas where MDA has not been supported by socio economic development, there is a persistent resurgence of the disease [4][page 210].

It has been demonstrated [19] that adult worms have a low probability of mating if the worm population density is very small (e.g. small W). A threshold worm population is needed to have a mating probability sufficient to sustain transmission. The mating probability also depends on the aggregation parameter κ. As $\kappa \to 0$, indicating high aggregation, the mating probability tends to 1 even at low worm burden as most worms are aggregated in a small number of individuals, increasing their probability of being mated. In contrast, lower aggregation (high κ values) of worms reduces the mating probability due to higher dispersion within the population. The worm mating probability of an adult female worm is defined in eqn 5 according to [19].

The effective reproduction number R_{eff} is estimated as in [19]. In this method, as the rates of change of the snail population are very fast compared to the rate of change of the adult worm population, we make the assumption that the snail populations reach their equilibrium i.e. $\frac{dS}{dt} = \frac{dE}{dt} = \frac{dI}{dt} = 0$. Applying these conditions to equations 3 , 2 and 1 in that order, we get:

$$\begin{aligned}
\frac{dI}{dt} &= 0 \\
\sigma E^* - (\mu_N + \mu_I)I^* &= 0 \\
I^* &= \Big(\frac{\sigma}{\mu_N + \mu_I}\Big)E^*,
\end{aligned}$$

where I^* and E^* represent the equilibrium numbers of infected and exposed snails respectivel and letting $T_1 = \frac{\sigma}{\mu_N+\mu_I}$, then $I^* = T_1 E^*$.

$$\begin{aligned}
\frac{dE}{dt} &= 0 \\
\frac{1}{2}\beta W H \phi \gamma S^* - (\mu_N + \sigma)E^* &= 0 \\
E^* &= \frac{\frac{1}{2}\beta W H \phi \gamma}{\mu_N + \sigma} S^*
\end{aligned}$$

Next, setting $T_2 = \frac{\frac{1}{2}\beta W H \phi \gamma}{\mu_N+\sigma}$, then $E^* = T_2 S^*$. And rewriting the equation for the equilibrium number of infected snails;

$$\begin{aligned}
I^* &= T_1 E^* \\
&= T_1 T_2 S^*
\end{aligned}$$

Finally, the total number of snails at equilibrium is:

$$\begin{aligned} N^* &= S^* + E^* + I^* \\ &= S^* + T_2 S^* + T_1 T_2 S^* \\ &= (1 + T_2 + T_1 T_2) S^* \end{aligned}$$

The equation for the susceptible snails then reduces to $\frac{dS}{dt} = 0$ which simplifies to:

$$\begin{aligned} f_N\Big(1 - \frac{N^*}{C}\Big)(S^* + E^*) - \mu_N S^* - \frac{1}{2}\beta W H \phi \gamma S^* = 0 \\ f_N\Big(1 - \frac{N^*}{C}\Big)(1 + T_2) - \mu_N - \frac{1}{2}\beta W H \Phi \gamma = 0 \end{aligned}$$

Substituting N^* in terms of S^* and rewriting the above equation, we get:

$$S^* = \frac{C(f_N(1 + T_2) - \mu_N - \frac{1}{2}\beta W H \phi)}{f_N(1 + T_2 + T_1 T_2)(1 + T_2)} \tag{19}$$

Incorporating the rate of change of worm burden given by equation 4 and substituting the equilibrium value of I^*, we get:

$$\begin{aligned} \frac{dW}{dt} &= \lambda I^* \rho - (\mu_W + \mu_H) W \\ &= \lambda T_1 T_2 S^* \rho - (\mu_W + \mu_H) W \\ &= (\mu_W + \mu_H) W \Big(\frac{\lambda T_1 T_2 S^* \rho}{(\mu_W + \mu_H) W} - 1 \Big) \end{aligned}$$

This is rearranged to get the following form for the rate of change of worm burden:

$$\frac{dW}{dt} = (\mu_W + \mu_H) W (R_{eff} - 1) \tag{20}$$

where R_{eff}, the effective reproduction number is defined as:

$$R_{eff} = \frac{\lambda \rho T_1 T_2 C (f_N(1 + T_2) - \mu_N - \frac{1}{2}\beta W H \phi \gamma)}{f_N(1 + T_2 + T_1 T_2)(1 + T_2)(\mu_W + \mu_H) W} \tag{21}$$

References

[1] J. C. Thomas, D. J. Weber, Epidemiologic methods for the study of infectious diseases, Oxford University Press, 2001.

[2] O. Diekmann, J. A. P. Heesterbeek, J. A. Metz, On the definition and the computation of the basic reproduction ratio r0 in models for infectious diseases in heterogeneous populations, Journal of mathematical biology 28 (4) (1990) 365–382.

[3] O. Diekmann, J. Heesterbeek, M. Roberts, The construction of next-generation matrices for compartmental epidemic models, Journal of the Royal Society Interface (2009) rsif20090386.

[4] M.-G. Basanez, R. M. Anderson, Advances in parasitology mathematical models for neglected tropical diseases: Essential tools for control and elimination, part a preface (2015).

[5] H.-P. Duerr, K. Dietz, M. Eichner, Determinants of the eradicability of filarial infections: a conceptual approach, Trends in parasitology 21 (2) (2005) 88–96.

[6] L. Berec, E. Angulo, F. Courchamp, Multiple allee effects and population management, Trends in Ecology & Evolution 22 (4) (2007) 185–191.

[7] R. M. May, Thresholds and breakpoints in ecosystems with a multiplicity of stable states, Nature 269 (5628) (1977) 471–477.

[8] J. Heesterbeek, O. Diekmann, Mathematical epidemiology of infectious diseases: model building, analysis and interpretation, Vol. 5, John Wiley & Sons, 2000.
